# A Rare Case of Cystic Hemolymphangioma Associated With Intestine Duplication

**DOI:** 10.7759/cureus.78197

**Published:** 2025-01-29

**Authors:** Nana Morishima, Rina Fujiwara-Tani, Ruiko Ogata, Shodo Sakai, Hiroki Kuniyasu

**Affiliations:** 1 Molecular Pathology, Nara Medical University, Kashihara, JPN; 2 Surgery, Nozaki Tokushukai Hospital, Daito, JPN

**Keywords:** abdominal cystic tumor, developmental disorder, hemolymphangioma, jejunal duplication, lymphangioma

## Abstract

Cystic hemolymphangioma and jejunal duplication are both typically congenital conditions and primarily identified in childhood, making adult diagnoses exceedingly uncommon. Cystic hemolymphangioma and intestinal duplication together are particularly rare. In this report, we present the case of an adult female patient with both. A pedunculated mass was identified outside the jejunal wall, with duplicated intestine on the stalk and cystic hemolymphangioma within the mass. Both lesions were surgically excised, and the patient has remained recurrence-free for five years postoperatively.

## Introduction

Cystic lymphangioma predominantly occurs in the head and neck region of children, with rare presentations in adults. This abnormality represents approximately 30% of abdominal cystic lesions [1,2]. Hemolymphangioma, characterized by the proliferation of both vascular and lymphatic components, is even less common [3]. Intestinal duplication, a congenital anomaly, can affect any segment of the gastrointestinal tract; however, adult cases are relatively rare [4]. The concurrence of these two conditions has been scarcely reported in the literature, with one case reported, which involved a child [5]. Herein, we present an exceptionally rare case of cystic hemolymphangioma and jejunal duplication in an adult woman.

## Case presentation

A 54-year-old woman presented to our hospital complaining of fatigue. Physical examination revealed hypertension (166/103 mmHg) but no other significant findings. Laboratory tests indicated mild liver dysfunction and hyperlipidemia, with no signs of inflammation (Table 1).

**Table 1:** Laboratory data *normal value; the Japanese Ministry of Health, Labour and Welfare has set no minimum standard value ST: aspartate aminotransferase; ALT: alanine aminotransferase; ALP: alkaline phosphatase; γGTP: gamma-glutamyl transpeptidase; AMY: serum amylase; LDH: lactate dehydrogenase; BUN: blood urea nitrogen; HDL-C: high-density lipoprotein cholesterol; CRP: C-reactive protein; FBS: fasting blood sugar

Parameters	Patient values	Reference range
RBC (10^4^/μL)	486	380-480
Hemoglobin (g/dL)	14.1	12-16
Hematocrit (%)	42.1	35-48
WBC (/μL)	6850	4000-8000
Platelets (10^4^/μL)	27	15-35
AST (U/L)	25	13-37
ALT (U/L)	31	8-45
ALP (IU/L)	297	206-477
Cholinesterase (U/L)	520	201-421
γGTP (U/L)	34	*30
AMY (U/L)	66	50-200
LDH (IU/L)	216	118-335
T*otal bilirubin* (mg/dL)	0.28	0.4-1.5
Total protein (g/dL)	7.2	6.5-8.0
Albumin (g/dL)	4.2	4.1-5.1
BUN (mg/dL)	14.7	8-20
Creatinine (mg/dL)	0.61	0.46-0.79
Uric acid (mg/dL)	5.4	2.6-5.5
Cholesterol (mg/dL)	249	142-220
Triglyceride (mg/dL)	681	30-149
HDL-C (mg/dL)	45	45-96
CRP (mg/dL)	0.15	*0.3
FBS (mg/dL)	101	70-109

**Figure 1:**
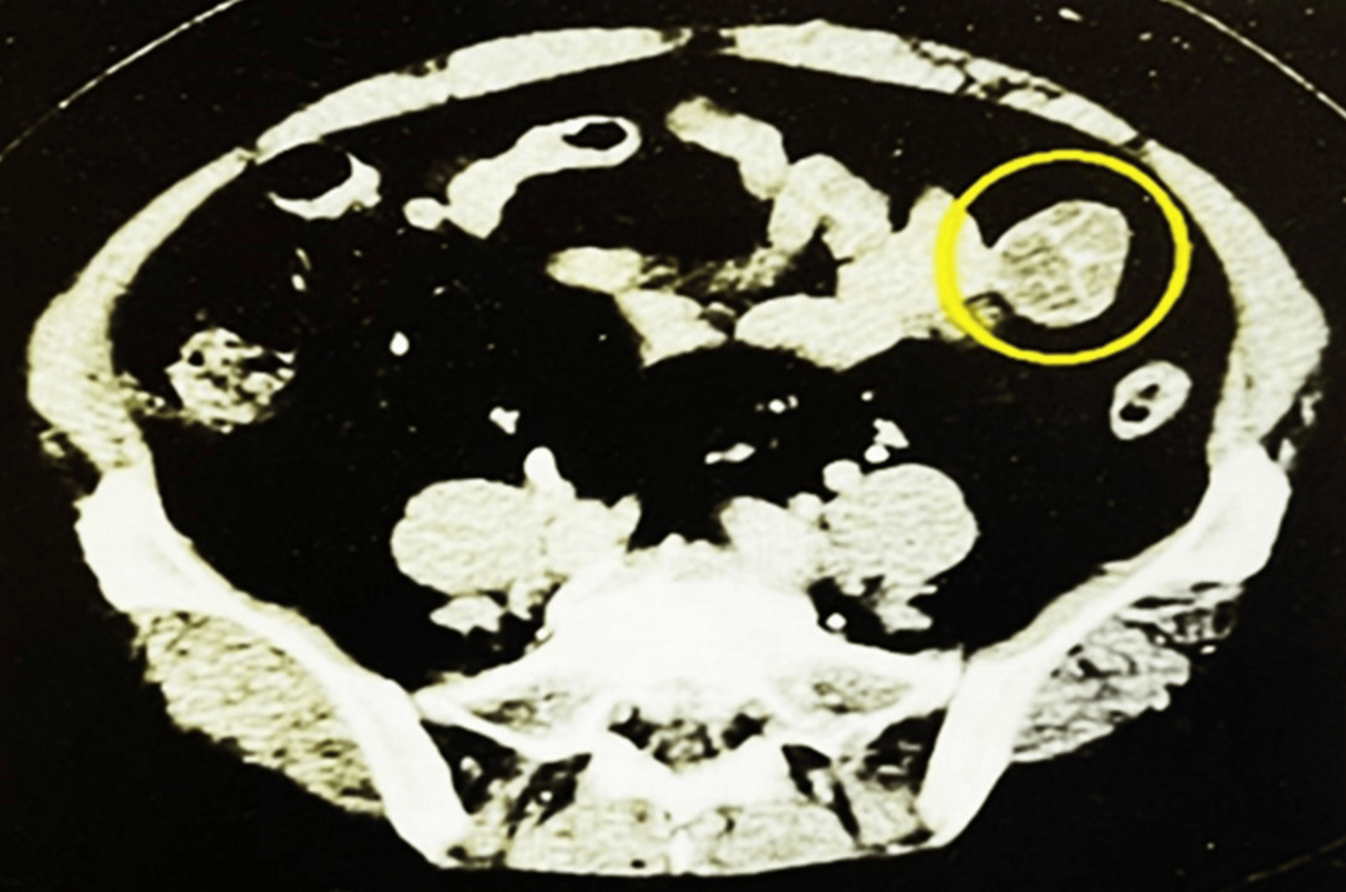
Abdominal computed tomography A protruding tumor is observed adjacent to the intestinal wall (circled). The intratumoral space is separated by septa containing low-density contents.

An abdominal ultrasound conducted a month later identified a 1.7-cm cyst in the liver lateral segment, alongside fatty liver changes. The patient visited the outpatient clinic monthly, and an abdominal CT scan was performed at the patient's next visit one month later to rule out fatty changes in the liver and neoplastic disease. The CT scan revealed a 3-cm extramural tumor with low density in the left small intestine (Figure 1). Contrast-enhanced CT suggested the presence of septation within the tumor capsule; however, there was no enhancement of the mass itself, and no obvious metastatic lesions were detected. Mild fatty changes were observed in the liver parenchyma.

**Figure 2:**
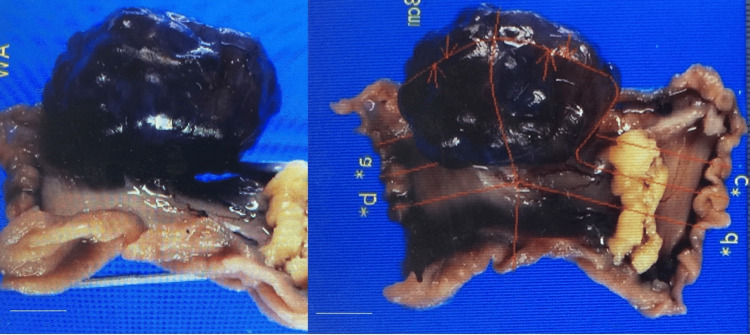
Macroscopic appearance of resected jejunum with tumor. A 4 cm cystic tumor is filled with serous fluid containing old blood and features a septum. The cystic tumor is attached to the jejunal serosa by a thin stalk. Scale bar: 1 cm.

As the condition did not require urgent treatment, one month later, for preventive purposes and to confirm the diagnosis of pathology, partial jejunal resection was undertaken for the excision of the small intestinal cystic tumor. A 4-cm cystic tumor was found protruding from the non-mesenteric side of the jejunum on a short stalk. The affected intestinal segment, including the tumor, was resected and anastomosed via a functional end-to-end technique. A simple hepatic cyst was found in the right lobe of the liver during surgery. Macroscopically, the jejunal cyst appeared multilocular with internal septa and contained serous fluid with signs of previous hemorrhage (Figure 2).

**Figure 3:**
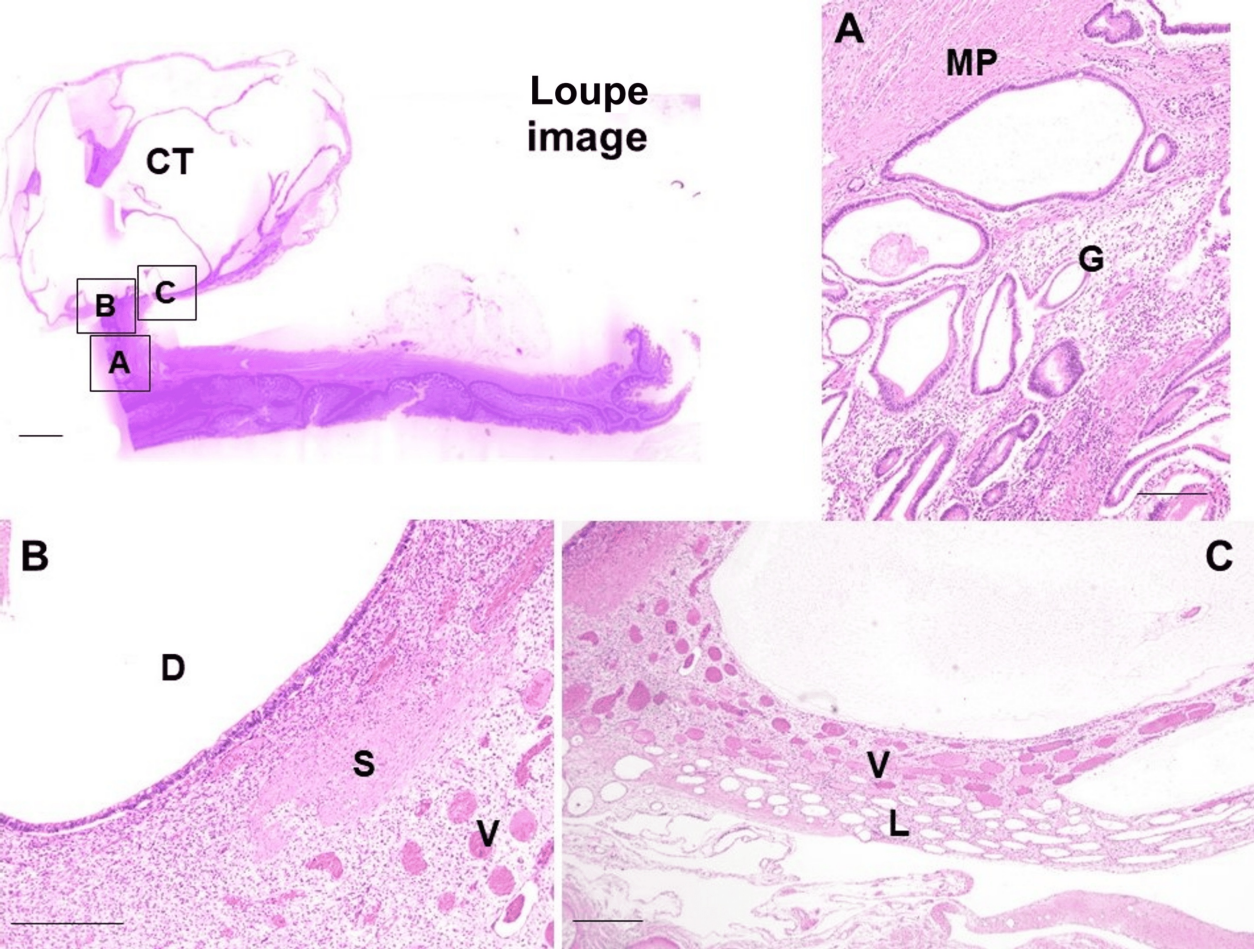
Histopathological appearance Loupe image showing a cystic tumor (CT) was bound to the jejunal serosa with a stalk. A. Jejunal subserosal layer showing glands covered with intestinal glandular epithelium (G), extending from the muscularis propria layer (MP) to the subserosal layer. B. Stalk portion displaying a dilated gland (D) surrounded by smooth muscle bundles (S), with blood vessel capillaries observed (V). C. Cystic tumor illustrating clusters of blood vessel capillaries and lymphatic vessel capillaries within the septum. Scale bar: 500 µm.

Histological examination revealed small glandular ducts lined by mucosal epithelium, extending from the jejunal wall to the tumor stalk (Figure 3A). The largest dilated duct, measuring 3 mm in diameter, was located at the junction of the tumor and stalk, surrounded by a smooth muscle layer (Figure 3B). The cyst exhibited fibrous septa dividing it into cavities of varying sizes, with small blood-filled vascular spaces and lymphatic-like cavities devoid of blood evident within the septa (Figure 3C).

**Figure 4:**
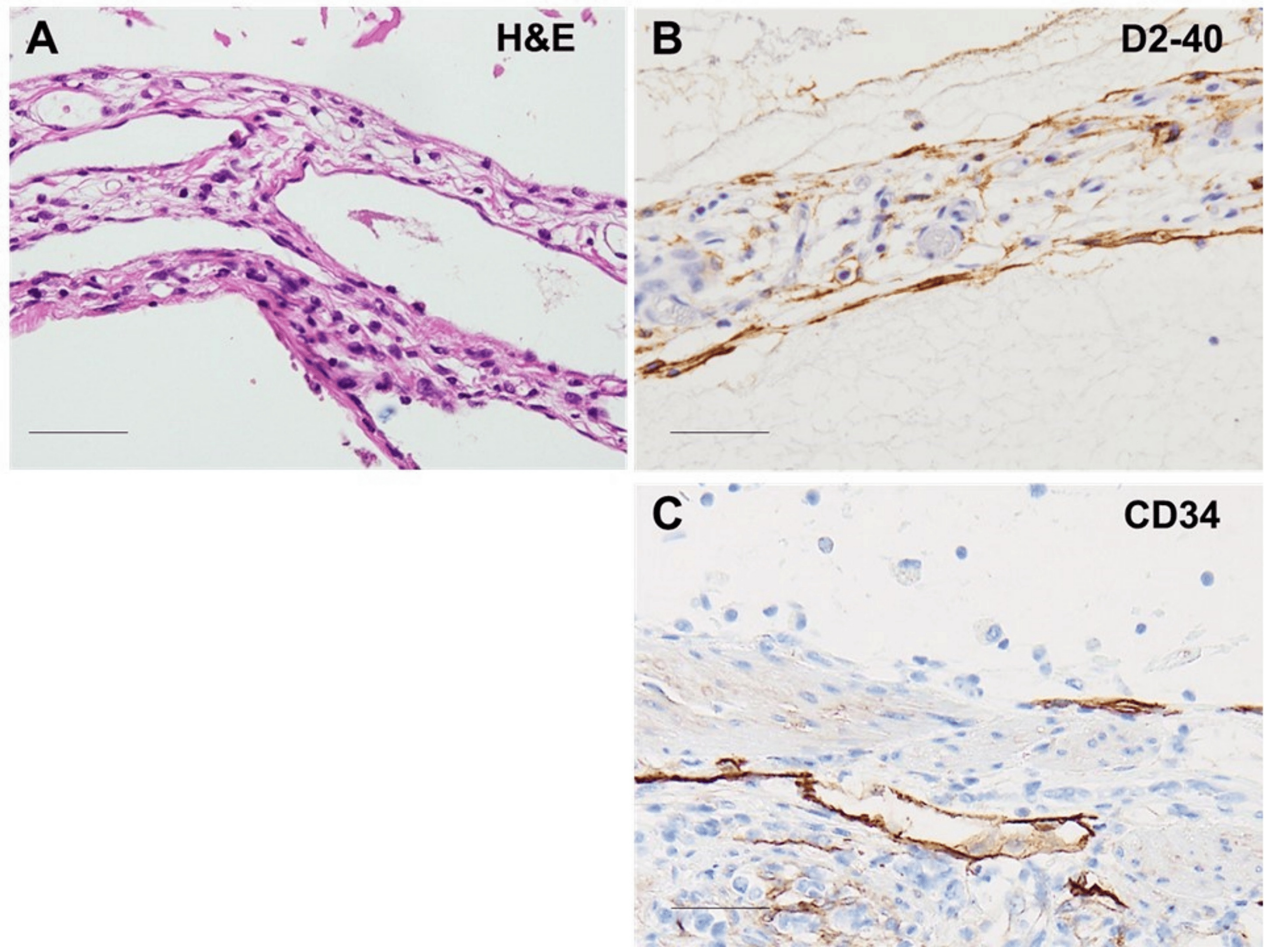
Immunohistochemistry of cystic tumor. A. H&E staining reveals lymphocyte infiltration in the septum. B. D2-40 staining shows D2-40-positive endothelial cells on the surface of the septum and intraseptal lymph vessel-like capillaries. C. CD34 staining shows CD34-positive endothelial cells in the surface of the intraseptal blood vessel-like capillaries. Scale bar: 50 µm.

The inner cyst lining and the endothelial-like cells of the small lumens of lymphatic capillaries tested positive for D2-40 immunostaining (Figure 4), while endothelial-like cells of capillary-like structures were positive for CD34.

Accordingly, the patient was diagnosed with jejunal cystic hemolymphangioma complicated by intestinal duplication. Post-surgical recovery was uneventful, and the patient was discharged on postoperative day 9. No recurrence was noted during the five-year follow-up.

## Discussion

In this case, a pedunculated cystic lesion was observed on the serosal surface of the jejunum. Differential diagnoses for peritoneal cystic lesions include cystic lymphangioma, cysts due to gastrointestinal duplication, ovarian cysts, mucinous cysts, mesenteric cysts, cystic mesothelioma, and gastrointestinal stromal tumors undergoing degeneration [6,7]. Of these lesions, only cystic hemolymphangioma is characterized by the presence of both D2-40-positive and CD34-positive endothelial-like cells lining the inner surface of the cyst, making it rarer than cystic lymphangioma [3,8].

Lymphangiomas most commonly present in the head and neck regions during childhood, with the highest incidence occurring in children under 10 years of age and in adults in their 40s. Less than 5% of lymphangioma cases occur in the abdomen, where they are typically found in the retroperitoneum (25%), mesentery (22%), and paracolic gutters (19%) [1]. When lymphangiomas do occur in the intestinal tract, they are most frequently located in the small intestine and colon [9]. The development of lymphangiomas can be attributed to congenital factors such as abnormal formation of lymphatic vessels during fetal development [10], as well as acquired factors such as lymphatic obstruction or congestion resulting from inflammation or trauma, including post-radiation changes [11,12].

Histopathologically, lymphangiomas are classified into three types: simple lymphangioma, cavernous lymphangioma, and cystic lymphangioma, with approximately 86% being multilocular cystic [8]. A cyst containing both blood and lymphatic vessels is classified as a hemolymphangioma. Histological diagnostic criteria include: (i) the lumen of the cyst is covered by flat endothelium rather than cuboidal or columnar epithelium, (ii) the presence of small lymphatic cavities in the cyst wall, (iii) lymphocyte clusters in the cyst wall, (iv) the presence of lipid-containing foam cells, and (v) the presence of smooth muscle in the cyst wall [13]. In this case, the cyst lumen was covered by (i) flat endothelium with elongated nuclei, and (ii) a lymphatic vessel-like lumen was present. The cyst inner wall showed (iii) lymphocytic infiltration, (iv) scattered foam cells, and (v) smooth muscle. Therefore, this case is considered to meet the five characteristics of cystic lymphangiomas.

Symptoms associated with lymphangiomas may include abdominal pain, abdominal distension, and palpable abdominal masses, with up to 16% of cases being asymptomatic [1]. Common complications include spontaneous or traumatic bleeding, rupture, and infection [14], while malignant transformation remains exceedingly rare [15].

In this case, we identified a glandular epithelium-covered lumen surrounded by a smooth muscle layer within the jejunal wall and the stalk of the cystic lesion, indicative of a duplicated intestinal segment. Gastrointestinal duplication is a rare congenital anomaly, predominantly observed in children, and reports of such duplications in adults are scarce [4]. Gastrointestinal duplication can occur anywhere along the gastrointestinal tract, from the esophagus to the anus, and is characterized by three key features: (i) the lesion is encased by one or more layers of smooth muscle, (ii) its inner surface is covered by gastrointestinal epithelium, and (iii) it is located adjacent to a segment of the native gastrointestinal tract [16]. The small intestine, particularly the ileocecal region, accounts for approximately 60% of these cases [17]. Intestinal duplications are primarily diagnosed in childhood, with about 70% detected in this age group, most commonly in the small intestine. The typical initial symptom is abdominal pain and preoperative diagnoses often misidentify the condition as intussusception, tumors, or intestinal obstruction, with only around 10% diagnosed as intestinal duplication [17].

In our case, the findings aligned with the defining characteristics of intestinal duplication, as we observed: (i) smooth muscle bundles resembling the muscularis propria surrounding the gland in the stalk, (ii) a single-layer columnar epithelium, and (iii) proximity to the non-mesentery side of the jejunum. Although jejunal duplications are more common, their occurrence in adults remains rare, and notably, this case presented no subjective symptoms.

In this case, a peritoneal cystic hemolymphangioma was found to be associated with a duplication of the intestinal tract. Such duplications have been reported in conjunction with other malformations, including spinal anomalies and malrotation [4,18]. A literature search revealed only one prior case of a peritoneal cystic lymphangioma associated with intestinal duplication, which involved a six-year-old boy who had experienced vomiting for over four years [5].

In contrast, our patient was a 54-year-old woman who had been experiencing fatigue for about a month, initially attributed to hyperlipidemia and liver dysfunction. However, the presence of bleeding in the cyst may have contributed to her nonspecific symptoms. Both cystic lymphangiomas and intestinal duplications are often found in children, with incidental discoveries in adulthood being rare, as seen in this case. While some reports indicate associations with conditions such as inflammation, intestinal volvulus, and intussusception, this case was asymptomatic and lacked such findings.

Both conditions can occur secondary to trauma. The current patient had no history of injury, suggesting that the peritoneal cystic hemolymphangioma and intestinal duplication may be developmental abnormalities. Pathological findings confirmed the presence of a duplicated intestine at the base of the cystic tumor comprising hemolymphangioma. Lymphatic endothelium differentiates from CD34-positive vascular endothelium and subsequently loses CD34 expression [19]. In the current case, the endothelium covering the large cavity of the cyst was D2-40 positive, while the capillary structures exhibited a mixture of D2-40-positive, bloodless vessels and CD34-positive, blood-filled capillaries, consistent with hemolymphangioma. Unlike vascular endothelium, lymphatic vessels primarily rely on glycolysis and fatty acid oxidation for energy metabolism, and epigenetic changes can enhance the transcription of lymphatic vessel-related genes [20]. The mixed proliferation of blood vessels and lymphatic vessels suggests immaturity or poor integration during capillary formation, a common feature that aligns with the impaired differentiation observed in intestinal duplication.

## Conclusions

This was a rare case of an incidentally discovered cystic hemolymphangioma along with extra-jejunal intestinal duplication in an adult female patient. It is necessary to be vigilant about the possibility of asymptomatic lesions such as this even in adults when examining images.
